# Hydroxyapatite–Silicon Scaffold Promotes Osteogenic Differentiation of CGF Primary Cells

**DOI:** 10.3390/biology12040528

**Published:** 2023-03-30

**Authors:** Laura Giannotti, Benedetta Di Chiara Stanca, Paola Nitti, Francesco Spedicato, Fabrizio Damiano, Christian Demitri, Nadia Calabriso, Maria Annunziata Carluccio, Andrea Palermo, Franco Ferrante, Luisa Siculella, Eleonora Stanca

**Affiliations:** 1 Department of Biological and Environmental Sciences and Technologies, University of Salento, 73100 Lecce, Italy; 2 Department of Engineering for Innovation, Campus Ecotekne, University of Salento, Via per Monteroni, 73100 Lecce, Italy; 3 National Research Council (CNR) Institute of Clinical Physiology (IFC), 73100 Lecce, Italy; 4 Implant Dentistry College of Medicine and Dentistry, Birmingham B4 6BN, UK; 5 Specialist in Oral Surgery, Private Practitioner, 73100 Lecce, Italy

**Keywords:** blood-derived biomaterials, CGF, growth factors, osteogenic differentiation, hydroxyapatite–silicon scaffold, tissue regeneration

## Abstract

**Simple Summary:**

The aim of this study was to identify new and innovative strategies to improve the tissue-regeneration process. Concentrated growth factor (CGF) is an autologous biomaterial rich in growth factors and multipotent stem cells. The purpose of our study was to evaluate the osteogenic differentiation of CGF primary cells in the presence of a hydroxyapatite–silicon scaffold, which represents a very interesting material in the field of bone reconstructive surgery. Our findings showed that the hydroxyapatite–silicon scaffold provided support to primary CGF cells by enhancing osteogenic differentiation. These data suggest interesting perspectives in the use of CGF together with scaffolds in the field of regenerative medicine.

**Abstract:**

The application of scaffolding materials together with stem cell technologies plays a key role in tissue regeneration. Therefore, in this study, CGF (concentrated growth factor), which represents an autologous and biocompatible blood-derived product rich in growth factors and multipotent stem cells, was used together with a hydroxyapatite and silicon (HA-Si) scaffold, which represents a very interesting material in the field of bone reconstructive surgery. The aim of this work was to evaluate the potential osteogenic differentiation of CGF primary cells induced by HA-Si scaffolds. The cellular viability of CGF primary cells cultured on HA-Si scaffolds and their structural characterization were performed by MTT assay and SEM analysis, respectively. Moreover, the matrix mineralization of CGF primary cells on the HA-Si scaffold was evaluated through Alizarin red staining. The expression of osteogenic differentiation markers was investigated through mRNA quantification by real-time PCR. We found that the HA-Si scaffold was not cytotoxic for CGF primary cells, allowing their growth and proliferation. Furthermore, the HA-Si scaffold was able to induce increased levels of osteogenic markers, decreased levels of stemness markers in these cells, and the formation of a mineralized matrix. In conclusion, our results suggest that HA-Si scaffolds can be used as a biomaterial support for CGF application in the field of tissue regeneration.

## 1. Introduction

Autologous platelet concentrates (APC) play a crucial role in tissue regeneration, since they are involved in cell proliferation and matrix remodeling, being rich in growth factors [1,2].

In recent decades, various techniques have been developed for the preparation of APC, classified into three different generations, as reported in [3,4,5,6,7]. The latest generation is concentrated growth factor (CGF), which contains higher levels of platelets and platelet-derived growth factors than other previous platelet derivatives [5,8]. The method to obtain CGF consists of venous blood collections that are centrifuged at alternating speeds, as set on the Silfradent device [9,10]. One of the main differences between CGF and most platelet preparations is that the production of CGF does not require the use of platelet and fibrin polymerization activators, since the CGF itself polymerizes slowly during centrifugation in a way similar to natural polymerization [11]. The technique of CGF preparation allows for obtaining a dense fibrin matrix, which guarantees a gradual release of growth factors and traps various cellular components including multipotent cells [1,2]. Our recent study reported that CGF has a fibrin structure containing primary cells that is positive for CD34, CD45, and CD105 surface markers of hematopoietic and mesenchymal stem cells [2]. Furthermore, some recent discoveries showed that CGF increased the proliferation of fibroblasts, endothelial cells, and osteoblasts thanks to the release of growth factors, suggesting that CGF could support the regeneration process [2,12,13]. CGF has been shown to induce osteogenic differentiation of human bone marrow stem cells (hBMSCs) by increasing the expression of RUNX2 (runt-related transcription factor 2, a key regulator of osteogenesis), COL1a1 (collagen Type I Alpha 1), and OCN (osteocalcin), which are well-known osteogenic markers [10]. In addition, culturing stem cells with CGF and biomaterials showed remarkable results in bone regeneration [10,14].

Multipotent stem cells derived from CGF seem to play a critical role in vasculogenesis, a crucial process in tissue regeneration [15,16]. Calabriso et al. showed that CGF released bone marrow-derived endothelial progenitor (EPC)-like cells, which contributed to neo-angiogenesis, the formation of endothelial tubular structures, by producing pro-angiogenic factors. Thus, CGF can be a reservoir of pro-angiogenic factors capable of inducing an angiogenic phenotype in mature endothelial cells. Overall, these data may suggest that peripheral blood could be an alternative source of stem/progenitor cells for clinical applications [13].

In several tissue-regeneration studies, the cell therapy based on different cell lines, such as embryonic stem cells and mesenchymal stem cells (MSC), has been reported [17,18]. To improve and support cell therapy, tissue engineering makes use of biocompatible scaffolds that promote both osteoconduction and osteoinduction [11]. The best scaffold should be biocompatible and have good integration in the bone tissue. Therefore, the scaffold should have an interconnected and highly porous 3D structure allowing for cell migration, vascularization, and diffusion of nutrients [19]. Studies have shown that porous scaffolds are ideal candidates for bone regeneration [20].

In the literature, studies on the biological and mechanical characteristics of different scaffolds incubated with hBMSC have been reported. A study by Kim et al. showed a greater biocompatibility of sintered HA substituted with magnesium and silicon, suggesting that it could be a useful material for bone augmentation [21]. Moreover, hBMSCs have been incubated with hydroxyapatite (HA), hydroxyapatite–magnesium (HA-Mg), hydroxyapatite–silicon (HA-Si), and hydroxyapatite–magnesium–silicon (HA-MgSi) scaffolds [22]. These investigations revealed that all scaffolds were biocompatible. Furthermore, due to their strong osteogenic potential on mesenchymal stem cells, HA-Si and HA-MgSi scaffolds could have some interesting applications in bone regeneration [22].

The use of human derived CGF together with scaffolds has not been extensively reported. Few studies available in the literature found that the combination of rabbit venous blood CGF, organic–inorganic bone biomimetic scaffolds, and nano-HA hybrid scaffold improved the proliferation and osteogenic differentiation of BMSCs in vitro and repaired in vivo damaged bone in rabbits [23]. Furthermore, Wang et al. have reported the use of n-hydroxyapatite, obtained from the *Ostrea cucullate* mollusk, as a scaffold material in the reconstruction of bone defects combined with CGF obtained from rabbit venous blood. The implantation of this biomaterial allowed for the observance of regeneration in the previously damaged tissue [24]. Thus, the aim of the present study was to investigate the effect of HA-Si scaffolds in promoting proliferation, adhesion, and osteogenic differentiation of human-derived CGF primary cells in vitro for use as a biomaterial support for CGF application in the field of tissue regeneration.

## 2. Materials and Methods

### 2.1. HA-Si Scaffold Fabrication and Characterization

The bioceramic scaffolds in silicon-doped hydroxyapatite (HA-Si) were obtained as previously reported in [22,25].

Briefly, powders of HA doped with 2% of Si were synthetized using calcium hydroxide Ca(OH)_2_, phosphoric acid (H_3_PO_4_ 85% *w*/*w*), and silicon tetraethyl orthosilicate (TEOS) Si(OC₂H₅)₄ (Sigma Aldrich, Milan, Italy) as precursors. The Ca/(P + Si) ratio was fixed at 1.67, with reference formula Ca₁₀(PO₄)_0.53_(SiO_4_^4-^)_0.07_(OH)₂. An amount of 0.53 M phosphoric acid was dissolved in distilled water, with ammonia (NH_3_) added to obtain a pH value of 10. Then, 0.07 M of hydrolyzed TEOS was added (using HNO_3_ as a catalyst), and the whole was stirred. A 1 M Ca (OH)_2_ solution was added, and the reaction mixture was stirred, maintaining the pH at 10.0 by adding NH_3_. Subsequently, the solution was precipitated and then placed in an oven at 100 °C for 24 h. After drying, the powders were crushed in a planetary mill and finally calcined at 900 °C.

Scaffolds were produced through a sponge replica method using polyurethane sponges with a density of 30 kg/m^3^ and cut into cubes with a volume of 1 cm^3^. The cubes were impregnated with a ceramic suspension obtained by adding 70% *wt* HA–Si 2% powders in a PVA 2% *wt* aqueous solution with an organic deflocculant (Dolapix CE-64, 0.5% by weight with respect to the ceramic powder), gently squeezed, and finally dried at 60 °C for 2 h. The sponges obtained were heated at a speed of 0.5 °C/min to 500 °C for 1 h to burn the polyurethane and then to 1300 °C with a speed of 1 °C/min for the sintering phase.

The macro and micro structures of HA-Si scaffolds were studied using a scanning electron microscope (SEM EVO R 40, Carl Zeiss AG, Oberkochen, Germany) with an accelerating voltage of 20 kV [26]. The percentage of weight loss under simulated physiological conditions up to 28 days and the corresponding mechanical properties of the ceramic scaffolds were evaluated to assess the biomaterial degradation [27,28]. The scaffolds were soaking in 50 mL of TRIS-HCl buffer (Trizma base 0.05 M, NaCl 0.15 M, Sodium azide 0.01% *w*/*v*, pH 7.4) at 37 °C (Julabo GmbH, Seelbach, Germany) for 3, 7, 14, and 28 days. On scheduled days, scaffolds were washed, dried at 60 °C, weighed to calculate the percentage of weight loss (Equation (1)), and tested in compression mode (crosshead speed of 0.5 mm/min) using a standard testing machine (Lloyd LR5K instrument, Fareham Hants, UK) equipped with a 1 kN load cell.

W loss% = (W initial − W final) / W initial(1)

Data are represented as mean ± standard deviation (DS) of 6 different replicates.

For biological tests, HA-Si scaffolds were sterilized as reported in [25].

### 2.2. Preparation of CGF and Culture of CGF Primary Cells

Blood samples of 8 mL were taken via venipuncture from 8 (5 male and 3 female) nonsmoker donors in good health. Informed consent was obtained from the donors included in this study according to the Declaration of Helsinki. Tubes of blood were processed as described in [2].

CGF was washed twice with phosphate-buffered saline (PBS) and placed into a 6-well plate; covered with low-glucose DMEM (L-DMEM) medium supplemented with 10% FBS (fetal bovine serum), 100 IU/mL penicillin/streptomycin, and 2 mM L-glutamine; and incubated at 37 °C with 5% CO_2_. The medium was replaced every day for the first 3 days in order to eliminate red blood cells released by CGF. After three days, CGF was chopped into small pieces to improve the release of primary cells. A total of 8 HA-Si scaffolds were put in each well of a 12-well plate, and on the top of them 2 CGF cells chopped in pieces were added. HA-Si scaffolds with CGF were cultured with basal medium (BM, L-DMEM) for 21 days. Half a volume of culture medium was replaced with fresh medium three times a week.

### 2.3. Proliferation Assay

Cell proliferation assay was performed using the 3-[4,5-dimethylthiazol-2-yl]-2,5 diphenyl tetrazolium bromide (MTT) test at different time points (3, 14 and 21 days). MTT is a widely used colorimetric method for assessing cell metabolic activity, based on the ability of viable cells to convert MTT, a soluble yellow tetrazolium salt, into an insoluble purple formazan precipitate. The intensity of the staining is proportional to both the amount and vitality of the cells and can be measured spectrophotometrically. Scaffolds were transferred into new 24-well plates, and the MTT test was performed as described in [22,25].

The cells released from CGF in the wells of the plate without scaffolds were used for control cells (CTR). All experiments were performed in triplicate, and data are represented as mean ± SD of triplicate measurements.

### 2.4. Osteogenic Differentiation Process

CGF primary cells plated on HA-Si scaffolds were cultured in basal medium (BM) and incubated at 37 °C with 5% CO_2_. To induce osteogenic differentiation, some of them were cultured in osteogenic medium (OM, L-DMEM with 10% FBS, 100 IU/mL penicillin/streptomycin, 2 mM L-glutamine, 10 mM β-glycerophosphate, 100 μM ascorbic acid 2-phosphate), for 21 days, as reported in [2].

### 2.5. SEM Analysis

For SEM analysis, samples were fixed in 2.5% glutaraldehyde in 0.1 M sodium cacodylate buffer, pH 7.4, for 1 h at room temperature, followed by two PBS washings and then dehydration in scalar ethanol/water solutions (50%, 70%, 80%, 90%, and 100% ethanol, 5 min each), after which they were freeze-dried.

To observe the inner surface, the scaffold was cut as described in [2]. The samples were observed at 5000× magnification.

Three different conditions were analyzed by SEM:

- HA-Si scaffold not incubated in the presence of CGF pieces, used as a negative control;
- HA-Si scaffold incubated with CGF for 21 days in BM;
- HA-Si scaffold incubated with CGF for 21 days in OM.

### 2.6. Alizarin Red Staining

Alizarin red S stain (ARS) (Sigma-aldrich, Milan, Italy) solution was prepared as described in [10].

HA-Si scaffolds were incubated with CGF pieces in a 12-well culture plate in BM or OM for 21 days. As a control (CTR), undifferentiated primary cells released from CGF were seeded in a 12-well culture plate in BM. Culture medium was changed at a rate of 50% 3 times a week.

ARS of CGF primary cells was performed at 21 days to detect osteoblast calcification. ARS was quantitated spectrophotometrically by adding 10% cetylpyridinium chloride [29]. Absorbance was measured at 562 nm by a spectrophotometer (Beckman Coulter DU800, Brea, CA, USA).

Data are represented as mean ± SD of triple measurements from three independent experiments.

### 2.7. DNA Quantification

Total DNA was used to normalize data due to its strong linear correlations with cell number present in the samples [30]. Scaffolds were grinded using a glass homogenizer; after that, DNA was extracted with HiPure Tissue DNA Mini kit (Guangzhou Meiji Biotechnology Co., Ltd., Guangzhou, China), according to the manufacturer’s protocol [31]. The DNA concentration was quantified using a UV spectrophotometer (ND-1000; NanoDrop Technologies; Thermo Fisher Scientific, Inc., Monza, Italy).

### 2.8. Real-Time PCR

Gene expression has been evaluated by real-time PCR in CGF primary cells cultured in the following experimental conditions:

- Undifferentiated CGF primary cells, used as a negative control;
- CGF primary cells grown on HA-Si scaffolds for 21 days in BM;
- CGF primary cells grown on HA-Si scaffolds for 21 days in OM.

Total RNA was extracted from 4 scaffolds for each condition, the reverse-transcriptase reaction was carried out, and then quantitative gene expression analysis was performed as described in [2]. Primers used in the real-time PCR are reported in Table 1. The efficiency of each primer was tested by running a standard curve in duplicate. The quantifications were performed using the ∆∆CT method, and the GAPDH gene was used as an internal control for normalization. The specificity of PCR products was confirmed by melting curve analysis.

Data are represented as mean ± SD of triple measurements from three independent experiments.

### 2.9. Statistical Analysis

Values were expressed as mean ± standard deviation (SD) for the indicated number of experiments. Differences between the two groups were settled as described in [2]. In all comparisons, *p* < 0.05 was considered statistically significant.

## 3. Results

### 3.1. HA-Si Scaffold Characterizations

**Figure 1:**
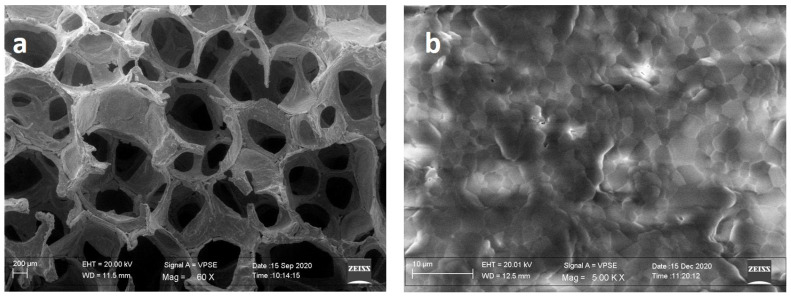
SEM image of HA-Si scaffold. (**a**) Macrostructure and (**b**) microstructure.

In the SEM micrograph (Figure 1a), the macro structure of the HA-Si scaffolds shows the highly interconnected cavities resulting from the PU combustion process. A well-defined grain structure with a densified microstructure with no visible defects is shown in Figure 1b.

**Figure 2:**
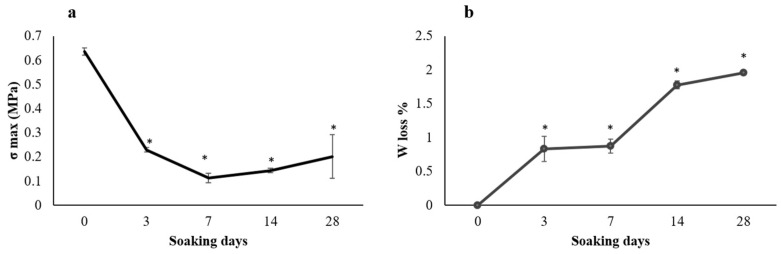
Plot of (**a**) mechanical strength and (**b**) weight loss% during soaking in Tris-HCl. Average values ± SD, n = 6. * *p* < 0.05 compared to 0 days.

Preliminary to the biological studies, the scaffold’s behavior in physiological conditions was studied, evaluating the weight loss and the related mechanical properties (Figure 2). After 3 days in Tris-HCl, the HA-Si scaffolds show a decrease in mechanical properties, which remain approximately unchanged up to 28 days. They show a constant weight loss for the first week and then progressively increase until the end of the incubation.

### 3.2. HA-Si Scaffold Biocompatibility for CGF Primary Cells

It has been reported that HA-Si scaffold has the best biocompatibility for the growth of hBMSCs [22]. Based on this study, we decided to test the HA-Si scaffold biocompatibility with CGF primary cells.

**Figure 3:**
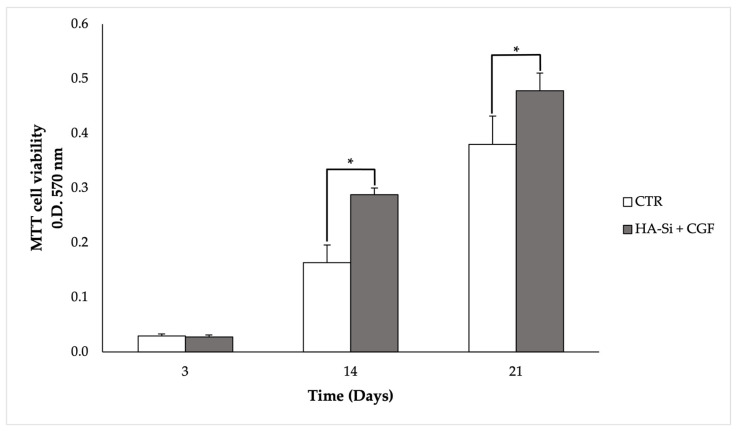
Proliferation and viability analysis of CGF primary cells cultured on HA-Si scaffold by MTT assay after 3, 14, and 21 days from seeding. CGF primary cells released directly on the plate were used as a control. Data are represented as mean ± SD of triplicate measurements from three independent experiments. * *p* < 0.05 compared to CTR, using Student’s *t*-test.

Figure 3 shows that after 3 days of culture, there is no significant difference between control and HA-Si scaffolds. However, the number of viable and metabolic active primary cells increases according to the increment of culture days. In fact, at 14 and 21 days, the optical density of the HA-Si scaffold significantly increased compared to the control sample. Therefore, these results show that the HA-Si scaffold has no cytotoxic effect and allows for the proliferation of cells adhering to its surface.

### 3.3. Effect of HA-Si Scaffolds on Matrix Mineralization of CGF Primary Cells

**Figure 4:**
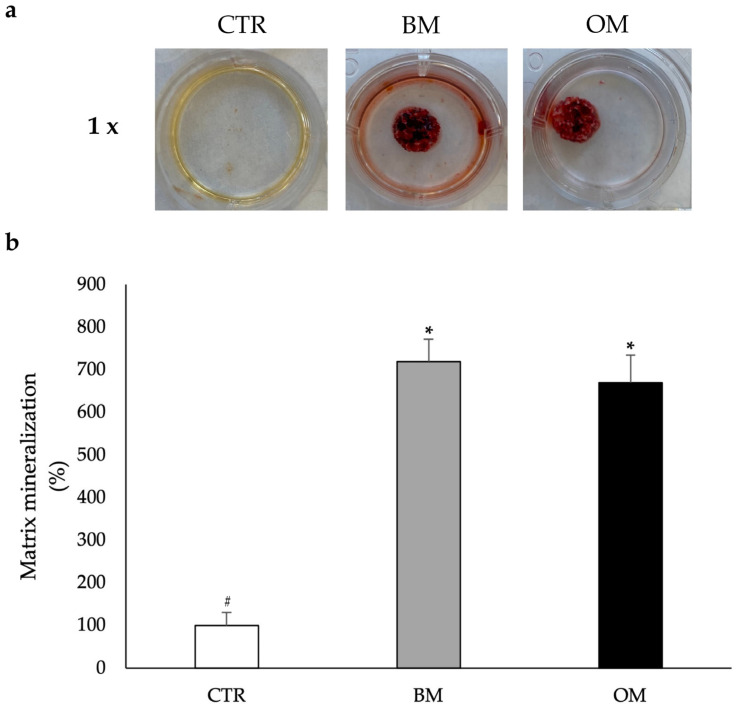
Osteogenic differentiation analysis of CGF primary cells grown on the HA-Si scaffold after 21 days of incubation. The CTR consists of CGF primary cells without HA-Si scaffold incubated in BM. Primary cells released from CGF were cultured on the scaffolds in BM or OM. (**a**) Photographs at 1× magnification. (**b**) Percentage of matrix mineralization of the three conditions normalized with DNA concentration. Data are represented as mean ± SD of triple measurements from three independent experiments. Samples bearing different symbols (#, *) differ significantly (*p* < 0.05; one-way ANOVA).

In order to measure the ability of the HA-Si scaffolds to induce the osteogenic differentiation of CGF primary cells, the Alizarin red assay was set up (Figure 4a). As reported, the use of an osteogenic medium causes the formation of a mineralized matrix in hBMSCs in vitro after 21 days of culture. In hBMSCs, the matrix mineralization requires the addition of substrates as BGP and AA [10,32].

Consequently, the scaffolds were cultured and incubated with CGF for 21 days. As reported in the histograms of Figure 4b, CGF primary cells grown on HA-Si scaffolds in BM show a significant increase in density values compared to the control. This increase is comparable to the one obtained with OM. These data suggest that in the presence of the HA-Si scaffolds, CGF primary cells are able to form a mineralized structure even in the absence of the inducers of the osteogenic process.

### 3.4. Effects of HA-Si Scaffolds on Osteogenic Differentiation Markers of CGF Primary Cells

To deeply examine at molecular level the osteogenic differentiation of CGF primary cells induced by the HA-Si scaffold, the expression of two osteogenic markers was quantified: RUNX2 and OCN. At the same time, the expression of characteristic stem cell markers was also quantified: CD105 and CD45.

**Figure 5:**
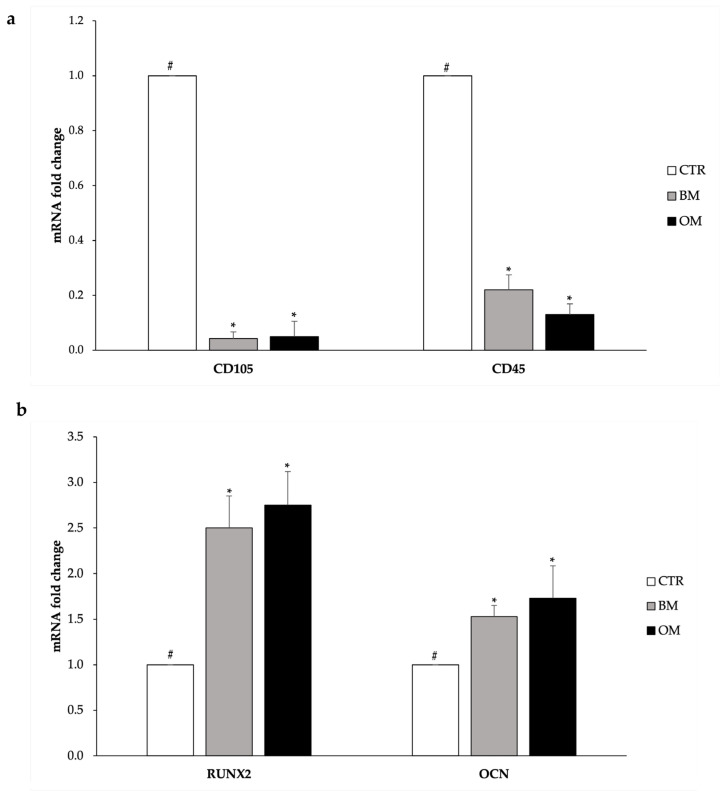
Analysis of the gene expression of the stemness markers (**a**) and osteogenic markers (**b**) of CGF primary cells. CTR: undifferentiated; CGF primary cells; BM: CGF primary cells grown on HA-Si scaffolds for 21 days in BM; OM: CGF primary cells grown on HA-Si scaffolds for 21 days in OM. GAPDH was used as a housekeeping gene for normalization. Results are expressed as mean ± SD of triplicate measurements from three independent experiments. Samples bearing different symbols (#, *) differ significantly (*p* < 0.05).

As regards the analysis of stem cell markers, the results reported in Figure 5a show that when compared to control cells, the expression of genes for CD105 and CD45 is significantly lower in cells grown on the HA-Si scaffold in terms of both the absence and presence of osteogenic differentiation inducers. In fact, the data obtained show that the abundance of mRNA for CD105 decreases by about 90% in cells cultured in either BM and OM; while the level of mRNA for CD45 records a 80% decrease in cells treated with BM and 87% in those treated with OM compared to control cells.

The expression levels of typical osteogenic differentiation genes can be observed in Figure 5b. RUNX2 mRNA level is significantly higher in the cells incubated with the HA-Si scaffolds compared to the control cells (+150% in the case of BM and +175% in OM). Considering the expression level of the OCN gene, its mRNA abundance is significantly higher in cells incubated with the scaffold in terms of presence or absence of differentiation inducers compared to the control cells (+53% in BM and +70% in OM).

### 3.5. Structural Characterization of CGF Primary Cells Grown on HA-Si Scaffolds

By means of SEM observation, it was possible to evaluate adhesion of the primary cells released by the CGF on the HA-Si scaffold.

**Figure 6:**
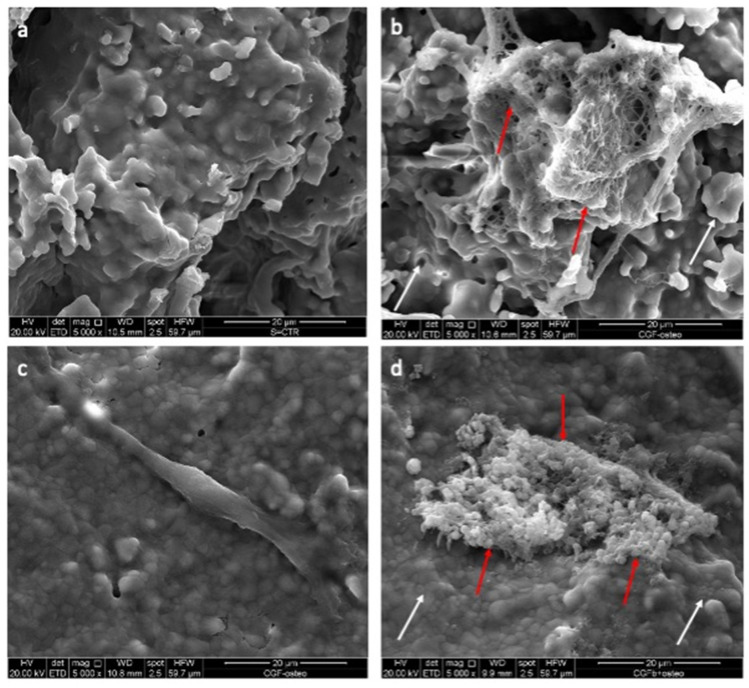
SEM images of CGF primary cells seeded on HA-Si scaffolds (scale bar = 20 μm). (**a**) HA-Si scaffold; (**b**) HA-Si scaffold cultured with CGF in BM for 21 days; (**c**) Cell released from CGF in BM for 21 days and adhered on HA-Si scaffold; (**d**) HA-Si scaffold cultured with CGF in OM for 21 days. White arrows indicate the HA-Si scaffold surface, red arrows indicate the mineralized structure formed by CGF primary cells after 21 days of incubation.

As shown in Figure 6c, the cells released by the CGF are able to adhere and colonize the interior of the HA-Si scaffold. Furthermore, in Figure 6b,c, it is possible to observe the formation of a cross-linked mineralized structure that is not present in Figure 6a, where it is represented on the HA-Si scaffold alone.

## 4. Discussion

In regenerative medicine therapies, blood-derived products have been used to obtain autologous stem cells instead of heterologous ones [2]. Various techniques of APC preparation have been developed to improve physiological processes of haemostasis and wound healing and to control inflammation processes [33]. Concentrates of growth factors and platelets derived from blood products have been used in various medical applications such as for ulcerative wounds and in implantology [34,35].

CGF, the third generation of platelet concentrates, plays an important role in the field of tissue regeneration. In order to analyze bone tissue regeneration, the effects of CGF on bone marrow stem cells (BMSC) was studied [11,36]. Recently, Rochira et al. [10], by analyzing ALP activity, an early osteogenic marker [37,38], have shown that after 14 days of treatment, CGF is able to induce an osteoinductive effect on hBMSCs, even better than that observed with OM-treated hBMSCs. Subsequently, by ELISA experiments, it has been demonstrated that CGF contains bioactive molecules, including growth factors such as vascular endothelial growth factor (VEGF), transforming growth factor- β1 (TGF-β1)), and metalloproteases (MMP-2, MMP-9), suggesting that CGF, thanks to the release over time of these bioactive molecules, can favor the tissue-regeneration process [2]. Furthermore, it has been shown that CGF also contains cells with two different morphologies, fusiform and spherical. The spindle cells express a high level of CD105 and CD45 surface markers and low levels of CD34 and do not express the mesenchymal markers CD73 and CD90; therefore, they do not appear as peripheral blood-derived mesenchymal stem cells but instead have their own characteristics [2]. Indeed, in the literature it has been reported that monocyte-derived cells express CD105, CD45, and CD14 and have the characteristics of mesenchymal cells and are able to separate into different cell lines [39].

In tissue engineering, an important role in regeneration could be played by scaffolds. The main characteristics of the scaffolds are nontoxicity to cells and interaction with surrounding tissue. Among the biocompatible materials most used are bioceramic scaffolds [40]. Therefore, our study analyzes bone regeneration promoted by the HA-Si scaffold on the primary cells of CGF.

The choice of HA-Si scaffolds has been justified by previous experiments demonstrating that they are good candidates in the study of tissue-regeneration processes. Cell proliferation experiments with hBMSCs and HA-Si scaffolds demonstrated that the scaffolds were biocompatible, allowing cell proliferation. Furthermore, the activity of ALP and the expression analysis of osteogenic markers (RUNX2 and OCN) revealed the ability of HA-Si scaffolds to promote osteoblast differentiation [22].

Characterization of the morphological and mechanical scaffolds was performed and showed that the scaffolds present a gradual increase in weight loss associated with loss in mechanical properties according to the increasing time of immersion in an aqueous solution. This is due to the biodegradability of Si-doped hydroxyapatite, causing a rapid loss of ions from the ceramic structure.

Subsequently, HA-Si scaffolds were incubated with CGF cut in pieces, and the biocompatibility of HA-Si scaffolds with CGF primary cells was tested. This analysis was set up by an MTT test to analyze cell viability at different time points (3, 14, and 21 days). The control condition was set up by using CGF primary cells released directly on the plate. By comparing the two conditions, an increase in cell proliferation was observed over time, suggesting that HA-Si scaffolds were noncytotoxic and ensured the proliferation of CGF primary cells.

Our previous study with ARS showed that CGF, in the presence of the osteogenic substrates BGP and AA, is able to induce a greater formation of mineralized matrix in hBMSCs compared to hBMSCs alone grown in osteogenic medium [10]. Therefore, an ARS experiment in which CGF was incubated with the HA-Si scaffold was set up. In this way, the ability of the HA-Si scaffold to induce osteogenic differentiation of CGF primary cells was measured. Our data show that after 21 days of incubation of CGF with the HA-Si scaffold, the primary cells released by CGF form a mineralized structure in the presence of osteogenic medium and in basal medium devoid of osteogenic substrates. This means that the scaffold itself can induce osteogenic differentiation by providing the calcium phosphates that CGF primary cells need to form a mineralized matrix; therefore, it is not necessary to add any other substrates.

A recent study has shown that the CGF primary cells left in culture with osteogenic medium for 3 weeks showed the formation of mineralized structures; the expression of the osteogenic markers RUNX2, COL1a1, and OCN; and loss of the stem cell markers [10].

Therefore, the next phase was to investigate the effects of HA-Si scaffolds on the expression of stemness or osteogenic differentiation markers of CGF primary cells. For this reason, the CGF was incubated for 21 days with the HA-Si scaffold in BM or OM, and after the incubation period, RNA was extracted from the primary cells released from CGF and adhered to the scaffolds. After 21 days of culture, the mRNA levels of stemness markers appear to be reduced in CGF primary cells in the presence or absence of the osteogenic medium compared to the control.

Consequently, after observing that CGF primary cells incubated with the HA-Si scaffold show a reduction in stemness markers (CD105 and CD45), the ability of these cells to differentiate into osteoblasts was also tested. The results reveal that the levels of mRNA for RUNX2 and OCN, markers of osteogenic differentiation, are increased in CGF primary cells incubated with the HA-Si scaffold compared to the control.

These data are congruent with the ability of silicon-substituted hydroxyapatite scaffolds to provide a strong osteogenic commitment to hBMSCs reported by Padmanabhan et al. [22]. However, the osteogenic medium we used is devoid of dexamethasone, the osteogenic differentiation inductor [41], and this could explain why the OM condition does not have an amplifying effect on scaffold-derived induction.

The morphological structure of the CGF primary cells grown on the HA-Si scaffold has been analyzed by SEM investigation. The cells released by the CGF adhered and colonized the inside of the HA-Si scaffold; however, after 21 days of osteogenic differentiation, they were probably incorporated in the mineralized matrix that they form and can be observed in the presence of CGF primary cells cultured both in BM and in OM.

The role of resident and circulating cells in tissue-regeneration processes is extensively studied [15,16], as is the characterization of the CGF, which contains and releases gradually over time growth factors and primary cells [2]. Therefore, we focused on the interactions that CGF can have with a biocompatible scaffold to induce cell differentiation and subsequently tissue regeneration. Our results reveal that HA-Si scaffolds could represent an excellent biomaterial in bone tissue engineering, being able, in combination with CGF, to create matrix mineralization and to induce primary cell differentiation without the use of any osteogenic medium. These results highlight the properties of CGF and its potential clinical applications.

## 5. Conclusions

The focus of this work was to highlight the possible use of HA-Si scaffolds as a biomaterial in supporting bone regeneration together with CGF. CGF acts as a reservoir of growth factors involved in regeneration and therefore is becoming one of the most useful strategies to enhance the regeneration response. Our results suggest that HA-Si scaffolds with CGF are able to promote osteogenic differentiation of CGF cell population without the aid of growth factors or other substrates.

## Figures and Tables

**Table 1:** Oligonucleotides used for real-time PCR analysis.

Gene Name	Accession Number	Sequences (5′-3′)	pb
PTPRC (CD45)	NM_080921.3	F: atgaccatgtatttgtggctta R: tgggggaaggtgttgggc	97
Endoglin (CD105)	NM_001278138.1	F: gccagcattgtctcacttca R: atgcgcaacaagctctttct	180
RunX2	NM_001278478.2	F: gacaaccgcaccatggtgg R: tctggtacctctccgaggg	160
OCN	NM_199173.6	F: gctacctgtatcaatggct R: cgatgtggtcagccaactc	111
GAPDH	AJ005371.1	F: atggccttccgtgtccccac R: acgcctgcttcaccaccttc	245

## Data Availability

The data presented in this study are available on request from the corresponding author. The data are not publicly available due to privacy.
